# A multidimensional follicular profile integrating oxidative stress, apoptosis and PI3K/AKT/mTOR signaling in relation to oocyte developmental competence in *in vitro* fertilization cycles

**DOI:** 10.1007/s10815-026-03909-y

**Published:** 2026-05-22

**Authors:** Oya Korkmaz, Seda Karabulut, Pelin Macit, İlknur Keskin

**Affiliations:** 1 Department of Histology and Embryology, Faculty of Medicine, Malatya Turgut Özal University, Malatya, Turkey; 2 Department of Histology and Embryology, International Faculty of Medicine, Istanbul Medipol University, Istanbul, Turkey; 3 In Vitro Fertilization Center, Istanbul Okan University Hospital, Istanbul, Turkey; 4 Department of Histology and Embryology, Faculty of Medicine, Istanbul Medipol University, Istanbul, Turkey

**Keywords:** Apoptosis, Follicular fluid, Oxidative stress, Oocytes, PI3K/AKT/mTOR signaling

## Abstract

**Purpose:**

Accurate assessment of oocyte developmental competence remains challenging in IVF, as morphology alone offers limited insight into intrinsic oocyte quality. Emerging evidence indicates that oocyte competence reflects coordinated regulation of the follicular microenvironment rather than a single biomarker. This study aimed to evaluate a multidimensional follicular marker panel integrating oxidative stress parameters, hormonal environment, granulosa cell apoptosis, chromatin integrity and PTEN/PI3K/AKT/mTOR signaling in relation to IVF outcomes.

**Methods:**

Thirty women undergoing ICSI were included, yielding a total of 211 oocytes. Individual follicles were analyzed according to oocyte maturity, embryo quality and pregnancy outcome. Follicular fluid oxidative stress parameters (TAC, TOC, OSI and MDA) and hormonal mediators (FSH, AMH, melatonin, TGF-β and β-hCG) were assessed. Granulosa cells were evaluated for DNA fragmentation, chromatin integrity and expression of PTEN/PI3K/AKT/mTOR pathway proteins.

**Results:**

Favorable IVF outcomes were associated with higher total antioxidant capacity and lower oxidative stress index and malondialdehyde levels, alongside reduced granulosa cell DNA fragmentation and improved chromatin integrity (*p* < 0.05). These outcomes were characterized by increased PI3K and AKT expression with concomitant mTOR suppression, while PTEN, PDK1, TSC1 and TSC2 levels remained unchanged. Follicular FSH and AMH were primarily linked to oocyte maturity, whereas melatonin, TGF-β and β-hCG were elevated in follicles yielding good-quality embryos and positive pregnancy outcomes (*p* < 0.05).

**Conclusions:**

Oocyte developmental competence in IVF reflects a coordinated follicular microenvironment integrating oxidative balance, granulosa cell integrity, hormonal regulation, and PI3K/AKT/mTOR signaling, supporting a multidimensional, panel-based framework for integrated follicular assessment.

**Graphical Abstract:**

**Figure:**
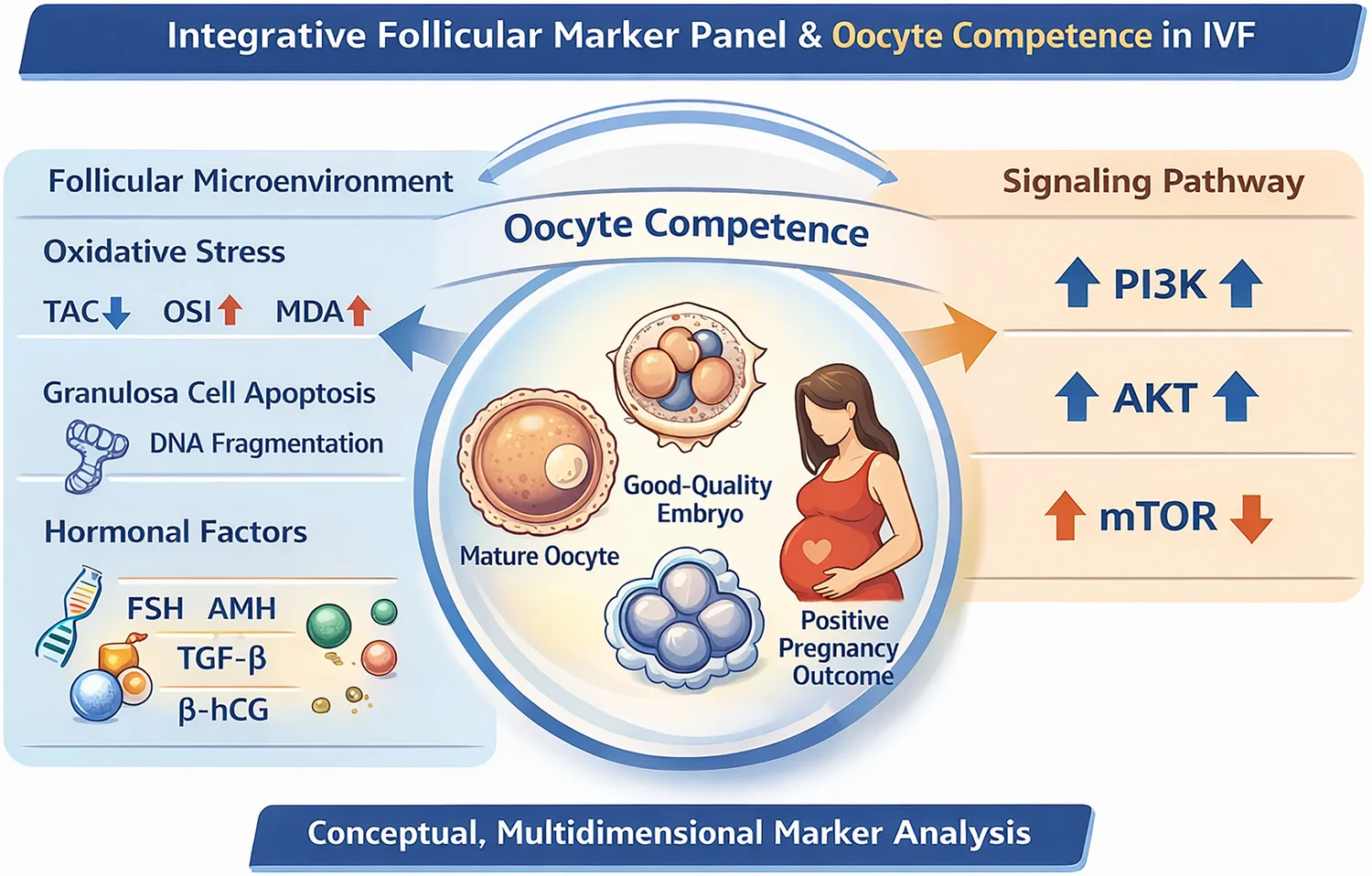

## Introduction

Despite major advances in assisted reproductive technologies (ART), the accurate assessment of oocyte developmental competence remains one of the most critical and unresolved challenges in in vitro fertilization (IVF). Oocyte competence, defined as the intrinsic ability of an oocyte to undergo maturation, fertilization, embryo development and ultimately support pregnancy, is a key determinant of IVF success [1]. Although controlled ovarian stimulation increases the number of retrievable oocytes, it also bypasses physiological follicular selection, resulting in oocytes with heterogeneous developmental potential [2, 3].

Currently, oocyte selection in IVF relies predominantly on morphological evaluation. However, morphological criteria alone provide limited insight into the molecular and functional status of the oocyte and fail to reliably predict developmental competence and pregnancy outcome [4, 5]. This limitation has driven increasing interest toward identifying non-invasive or minimally invasive biological markers that more accurately reflect oocyte quality.

The ovarian follicle constitutes a highly specialized microenvironment in which the oocyte develops in close interaction with surrounding granulosa and cumulus cells and the follicular fluid. Bidirectional communication within this microenvironment regulates oocyte growth, meiotic progression and cytoplasmic maturation [6, 7]. Consequently, the biochemical, molecular and cellular composition of the follicular milieu is increasingly recognized as a critical determinant of oocyte competence.

Among the factors shaping the follicular microenvironment, oxidative stress plays a central role. While physiological levels of reactive oxygen species (ROS) are essential for normal folliculogenesis and ovulation, excessive oxidative stress disrupts meiotic spindle integrity, damages DNA and impairs mitochondrial function, ultimately compromising oocyte quality [8]. Altered oxidative stress parameters in follicular fluid have been associated with reduced fertilization rates, impaired embryo development and poor IVF outcomes, although reported findings remain heterogeneous [9, 10]. These inconsistencies suggest that oxidative stress should not be interpreted in isolation but rather as part of a broader follicular context.

Another fundamental regulatory axis in follicular development is the PTEN/PI3K/AKT/mTOR signaling pathway. This pathway orchestrates primordial follicle activation, granulosa cell proliferation and survival, metabolic regulation and oocyte meiotic progression [11–13]. Experimental and clinical studies indicate that dysregulation of PI3K/AKT/mTOR signaling alters folliculogenesis and oocyte competence, yet its precise role in human IVF outcomes remains incompletely understood [14, 15]. Moreover, growing evidence suggests crosstalk between PI3K/AKT signaling, oxidative stress responses and DNA damage pathways, highlighting the integrated nature of follicular regulation [16, 17].

Granulosa cell integrity and apoptosis further represent critical components of the follicular environment. Increased apoptosis and DNA fragmentation in granulosa and cumulus cells have been associated with impaired oocyte maturation, reduced embryo quality and lower pregnancy rates in IVF cycles [7, 18]. Chromatin integrity within these cells reflects cellular health and may indirectly mirror the developmental potential of the enclosed oocyte.

In addition to oxidative and molecular signaling pathways, the hormonal composition of follicular fluid provides essential cues for follicular development. Follicular levels of hormones such as follicle-stimulating hormone (FSH), anti-Müllerian hormone (AMH), melatonin, transforming growth factor-β (TGF-β) and β-human chorionic gonadotropin (β-hCG) have been implicated in oocyte maturation, embryo development and implantation potential [19, 20]. However, no single hormonal marker has demonstrated sufficient predictive power when evaluated independently.

Collectively, these observations support the concept that oocyte developmental competence does not arise from a single determinant but rather from the coordinated regulation of oxidative balance, survival signaling, hormonal environment and granulosa cell integrity within the follicular microenvironment. Nevertheless, human studies simultaneously integrating these dimensions remain limited.

Therefore, the present study aimed to evaluate a multidimensional follicular marker panel encompassing oxidative stress parameters, hormonal mediators, granulosa cell apoptosis and chromatin integrity, together with PTEN/PI3K/AKT/mTOR pathway signaling, in relation to oocyte maturity, embryo quality and pregnancy outcomes in IVF cycles. By adopting a panel-based and pattern-oriented approach, this study seeks to provide a more comprehensive understanding of the follicular determinants associated with oocyte developmental competence.

By simultaneously evaluating multiple biological dimensions within the same follicular context, this study differs from previous single-marker approaches and provides an integrated evaluation of follicular features rather than advancing a novel mechanistic framework. Rather than representing exclusively positive determinants, the evaluated parameters reflect both favorable and unfavorable biological conditions that collectively influence oocyte quality within the follicular microenvironment.

## Materials and methods

### Ethics approval and consent to participate

Ethical approval for this study was obtained from the Istanbul Medipol University Non-Interventional Clinical Research Ethics Committee (Approval No: 10840098–604.01.01-E.63597/1041). All participants provided informed consent after being informed about the study objectives, voluntary nature of participation and data confidentiality. The study procedures were conducted in accordance with institutional ethical standards and the principles of the Declaration of Helsinki.

### Study design, study population and sample collection

This prospective observational study included 30 couples undergoing intracytoplasmic sperm injection (ICSI) at the Istanbul Çamlıca Medicana Hospital IVF Center between January and August 2020. Only first-time ICSI cycles were included. Female inclusion criteria were age 25–40 years, body mass index (BMI) 19–26 kg/m^2^, controlled ovarian stimulation using an antagonist protocol, retrieval of 5–15 mature (MII) oocytes, and primary infertility due to bilateral tubal obstruction confirmed by hysterosalpingography. Women diagnosed with polycystic ovary syndrome according to the Rotterdam criteria [21], endometriosis at any stage, or cycles requiring coasting were excluded.

Male partners aged 20–42 years with normal semen parameters (normozoospermia) and BMI 19–26 kg/m^2^ were included. Couples were excluded if either partner had anatomical or chromosomal abnormalities of the reproductive system, systemic disease, smoking or alcohol consumption, or regular medication use. Cycles involving adjunctive ART procedures (preimplantation genetic testing, assisted hatching, in vitro maturation or coculture) were excluded. To enable accurate attribution of outcomes to individual oocytes, only cycles with single embryo transfer were analyzed.

A total of 211 oocytes were obtained from 30 women and classified according to IVF parameters as follows: oocyte maturity [mature (n = 176) and immature (n = 35)], embryo quality [good quality (n = 71) and fair/poor quality (n = 140)], and pregnancy outcome [positive (n = 14) and negative (n = 16)].

Although the study was prospectively designed, analyses were performed at the individual follicle–oocyte level to identify coordinated biological associations rather than to establish predictive thresholds.

### IVF parameters

#### Controlled ovarian stimulation and oocyte retrieval

All women underwent controlled ovarian stimulation using a GnRH antagonist protocol with recombinant follicle-stimulating hormone (rec-FSH). The initial rec-FSH dose (125–600 IU) was individualized according to ovarian reserve and age. Ovulation was triggered with human chorionic gonadotropin (hCG; 5000 IU, Ovitrelle®, Merck Serono) when at least three follicles reached a diameter of ≥ 18 mm. Oocyte pick-up (OPU) was performed transvaginally under ultrasound guidance approximately 35.5 h after hCG administration using a single-lumen aspiration needle (Swemed, Sweden).

#### Follicular fluid and granulosa cell collection

Follicular aspirates were collected individually from each follicle during OPU. Cumulus–oocyte complexes were isolated under a stereomicroscope, and the remaining aspirates were processed for follicular fluid and granulosa cell separation. Samples contaminated with blood or aspiration medium were excluded. Follicular fluids were centrifuged at 600 g for 10 min, and the supernatant and cell pellets were separated. Granulosa cells and follicular fluid samples were stored at − 20 °C until analysis. Additional follicular cells obtained during denudation were washed and centrifuged under identical conditions and stored separately.

#### Oocyte denudation and evaluation

**Fig. 1:**
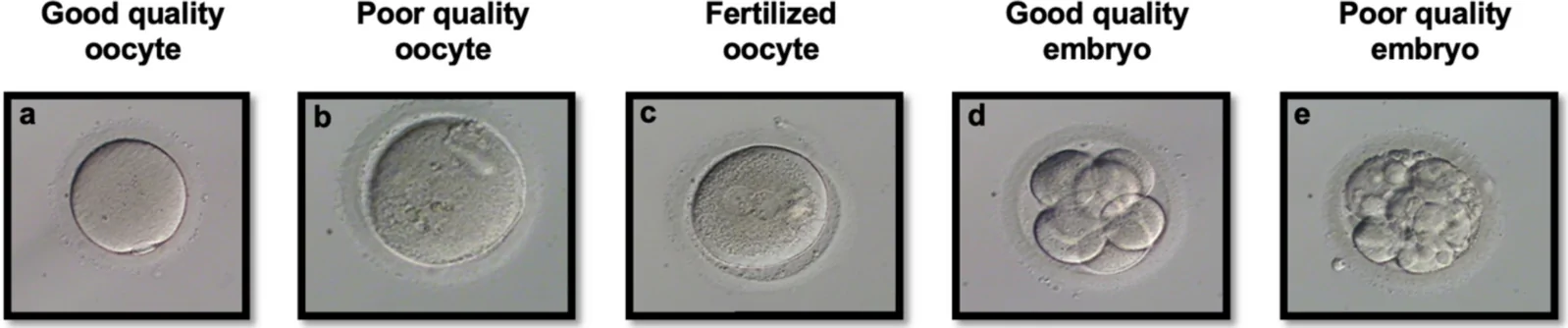
Morphological evaluation of oocytes and embryos. Representative light microscopy images illustrating morphological classification of oocytes and embryos. (**a**) Good-quality oocyte showing homogeneous and bright cytoplasm, normal zona pellucida, and an intact polar body. (**b**) Poor-quality oocyte characterized by granular cytoplasm, cytoplasmic vacuolization, fragmented polar body, and inclusion bodies. (**c**) Fertilized oocyte exhibiting two pronuclei and two polar bodies. (**d**) Good-quality embryo on day 3 with equal-sized blastomeres, absence of fragmentation, and homogeneous cytoplasm. (**e**) Poor-quality embryo on day 3 displaying marked fragmentation, unequal blastomeres, and thickened zona pellucida

Oocytes were denuded enzymatically using hyaluronidase (Hyase 10X, Vitrolife, Sweden) and mechanically by pipetting. Oocyte maturity and morphology were assessed under an inverted microscope at × 40 magnification. The presence of the first polar body indicated a mature oocyte arrested at metaphase II (MII), while its absence indicated an immature oocyte arrested at metaphase I (MI). Oocyte quality was classified based on cytoplasmic homogeneity, size, zona pellucida thickness and the absence of inclusions or vacuoles, as previously described (Fig. 1).

#### Semen preparation, ICSI and embryo culture

Semen samples were prepared using a two-layer density gradient method (90%/45%) as described by Yılmaz et al. [22]. Intracytoplasmic sperm injection (ICSI) was performed on MII oocytes and MI oocytes that matured within 4 h, according to the method of Van Steirteghem et al. [23]. Injected oocytes were cultured in microdroplets of G-IVF medium (Vitrolife) under oil at 37 °C in an atmosphere of 5% CO₂, 6% O₂ and 95% humidity. Fertilization was confirmed by the presence of two pronuclei and two polar bodies 16–18 h after injection.

Embryonic development was evaluated on days 2, 3 and 5. Embryo quality was graded according to the criteria of Staessen et al. [24], with Class A and B embryos considered good quality. Blastocyst development and grading were assessed on day 5 using the Gardner and Schoolcraft classification system [25].

#### Embryo transfer and pregnancy assessment

All patients underwent single embryo transfer on day 5 under ultrasound guidance using a Wallace catheter (Smiths, UK), following progesterone supplementation (Crinone® 8% gel, Merck Serono). Clinical pregnancy was defined as a serum β-hCG level ≥ 50 mIU/mL 12 days after transfer with appropriate doubling, followed by ultrasonographic visualization of a gestational sac one week later.

### Oxidative stress determination

Oxidative stress status in follicular fluid samples was assessed by measuring total antioxidant capacity (TAC), total oxidant capacity (TOC), oxidative stress index (OSI) and malondialdehyde (MDA) levels. TAC and TOC were determined using commercially available assay kits (Rel Assay Diagnostics, Sigma-Aldrich) according to the manufacturer’s instructions. OSI was calculated as the ratio of TOC to TAC using the formula:

$$OSI=TOC\left(\mu mol\;H_2O_2Eq/L\right)/TAC\left(\mu mol\;Trolox\;Eq/L\right)\times100$$

MDA levels, as an indicator of lipid peroxidation, were quantified using a thiobarbituric acid reactive substances (TBARS) spectrophotometric method. Following protein precipitation with trichloroacetic acid, samples were incubated with thiobarbituric acid and extracted with n-butanol. After centrifugation, absorbance of the organic phase was measured at 532 nm using a multimode microplate reader (BioTek Synergy HTX, BioTek Instruments, USA). MDA concentrations were calculated using an extinction coefficient of 1.56 × 10^5^ M⁻^1^·cm⁻^1^ and expressed as µmol/L.

All oxidative stress parameters were evaluated in relation to IVF outcome groups, including oocyte maturity, embryo quality and pregnancy outcome.

### Expression and localization of PTEN/PI3K/mTOR pathway proteins

Immunocytochemistry and immunofluorescence analysis were performed to evaluate the expression and cellular localization of PTEN/PI3K/mTOR signaling pathway proteins (PTEN, PI3K, PDK1, AKT, mTOR, TSC1 and TSC2) in follicular cells, including mural and cumulus granulosa cells.

#### Immunocytochemistry

Follicular cells were fixed in 4% paraformaldehyde, washed in phosphate-buffered saline (PBS) and treated with 3% hydrogen peroxide to block endogenous peroxidase activity. Antigen retrieval was performed using citrate buffer, followed by protein blocking. Slides were incubated overnight at 4 °C with primary antibodies against PTEN, PI3K, PDK1, AKT, mTOR, TSC1 and TSC2 at optimized dilutions [PTEN (1:500, Cat no. GTX101025, Genetex, USA), PI3K (1:500, Cat no. MA5-17150, Invitrogen, USA), AKT (1:400, Cat no. 60203–2-Ig, Proteintech, USA), mTOR (1:100, Cat no. AHO1232, Invitrogen, USA), PDK1 (1:500, Cat no. ab110025, Abcam, England), TSC1 (1:200, Cat no. 20988–1-AP, proteintech, USA), TSC2 (1:100, Cat no. B0590, Assaybiotech, USA)]. After washing, cells were incubated with a biotinylated secondary antibody and streptavidin–peroxidase complex, and immunoreactivity was visualized using diaminobenzidine (DAB). Nuclei were counterstained with Mayer’s hematoxylin. Stained cells were evaluated under a light microscope (Nikon, Japan), and immunopositivity was calculated as the percentage of positively stained cells by counting at least 50 cells per sample [26]. Image evaluation and cell counting were performed in a blinded manner to minimize observer bias.

#### Immunofluorescence analysis

Cell smears were fixed in 4% paraformaldehyde, subjected to antigen retrieval and blocked with normal goat serum containing Triton X-100. Slides were incubated overnight at 4 °C with primary antibodies against PTEN, PI3K, PDK1, AKT, mTOR, TSC1 and TSC2 [PTEN (1:200, Cat no. GTX101025, Genetex, USA), PI3K (1:100, Cat no. MA5-17150, Invitrogen, USA), AKT (1:100, Cat no. 60203–2-Ig, Proteintech, USA), mTOR (1:200, Cat no. AHO1232, Invitrogen, USA), PDK1 (1:200, Cat no. ab110025, Abcam, England), TSC1 (1:100, Cat no. 20988–1-AP, Proteintech, USA), TSC2 (1:200, Cat no. B0590, Assaybiotech, USA)], followed by incubation with Alexa Fluor 488- or 568-conjugated secondary antibodies. Nuclei were counterstained with DAPI, and slides were mounted using antifade medium [26]. Fluorescent signals were examined using a confocal microscope (Zeiss LSM 800, Carl Zeiss).

All primary antibodies were validated according to the manufacturer’s specifications, and appropriate positive and negative controls were included.

### Biochemical analysis of follicular fluid

Follicular fluid concentrations of follicle-stimulating hormone (FSH), anti-Müllerian hormone (AMH), melatonin, transforming growth factor-β (TGF-β), and β-human chorionic gonadotropin (β-hCG) were measured using enzyme-linked immunosorbent assay (ELISA) kits according to the manufacturers’ instructions.

The following commercially available kits were used: FSH (Cat no. E-EL-H1143), AMH (Cat no. E-TSEL-H0018), melatonin (Cat no. E-EL-H2016), TGF-β (Cat no. E-EL-0162), and β-hCG (Cat no. E-EL-H3647) (all from Elabscience, China).

Hormonal levels were analyzed in relation to IVF outcome parameters, including oocyte maturity, embryo quality, and pregnancy outcome. All ELISA assays were performed according to the manufacturer’s instructions, and assay sensitivity and detection ranges were within the specified limits.

### Apoptosis markers (DNA fragmentation and chromatin integrity)

#### DNA fragmentation assessment

We assessed DNA fragmentation in granulosa cells using TUNEL staining with the In Situ Cell Death Detection Kit (Cat no. 11684795910, Roche, Indianapolis, USA) according to the manufacturer’s instructions. In summary, smears were fixed with 4% paraformaldehyde. After fixation, it was washed with PBS and incubated on ice with a 0.1% Triton X-100 solution for permeabilization. It was then incubated with TUNEL reaction solution at 37 °C. After incubation, PBS was washed. In order to make the cell nuclei visible, the preparations were covered with a coverslip using a fluoromount medium containing DAPI. TUNEL (+) and TUNEL (-) cells were analysed with a Zeiss LSM 800 confocal microscope (Carl Zeiss) in at least 50 cells from each patient in each group [26]. Outcomes according to IVF parameters (oocyte maturity, embryo quality, and pregnancy) were evaluated.

#### Chromatin integrity assessment

Chromatin integrity was assessed by Toluidine Blue (TB) staining. TB staining was selected as a practical and well-established method for evaluating chromatin integrity and DNA packaging status through its affinity for DNA phosphate groups. Although it has been widely used in sperm chromatin assessment, its application in somatic cells, including granulosa cells, has also been reported as an indicator of nuclear integrity and cellular damage, as demonstrated in both the literature and our prior work [26]. Smears were fixed in a 96% ethanol-acetone (1:1) mixture at 4 °C. Then hydrolysis was carried out in 0.1N HCl at 4 °C and washed with distilled water. After washing, cells were stained with 0.05% Toluidine Blue for 5 min and washed again with distilled water. After washing, they were kept in 96% alcohol to remove the water, and the preparations were covered with a coverslip using a closure medium. Pale blue-stained cells were evaluated as TB (-). Dark blue or purple-stained cells were evaluated as TB (+). The percentage of stained and unstained cells (%) was calculated by counting at least 50 cells in the follicular cells obtained from the follicles of each patient in each group under a light microscope [26]. Outcomes according to IVF parameters (oocyte maturity, embryo quality, and pregnancy) were evaluated.

### Statistical analysis

We performed statistical analyses using IBM SPSS Statistics software (version 25.0; IBM Corp., Armonk, NY, USA). Normality of continuous variables was assessed using the Shapiro–Wilk test. Data were expressed as mean ± standard deviation (SD) for normally distributed variables and as median (interquartile range) for non-normally distributed variables.

Comparisons between two independent groups were performed using the independent samples t-test or Mann–Whitney U test, as appropriate. Categorical variables were analyzed using the chi-square test. Analyses were conducted according to predefined IVF outcome parameters, including oocyte maturity, embryo quality and pregnancy outcome.

A two-sided *p* value < 0.05 was considered statistically significant. Effect sizes were calculated to estimate the magnitude of group differences, using Cohen’s d for normally distributed variables and rank-biserial correlation coefficients (r) for non-normally distributed variables.

## Results

### Oxidative stress parameters in relation to IVF outcomes

**Fig. 2:**
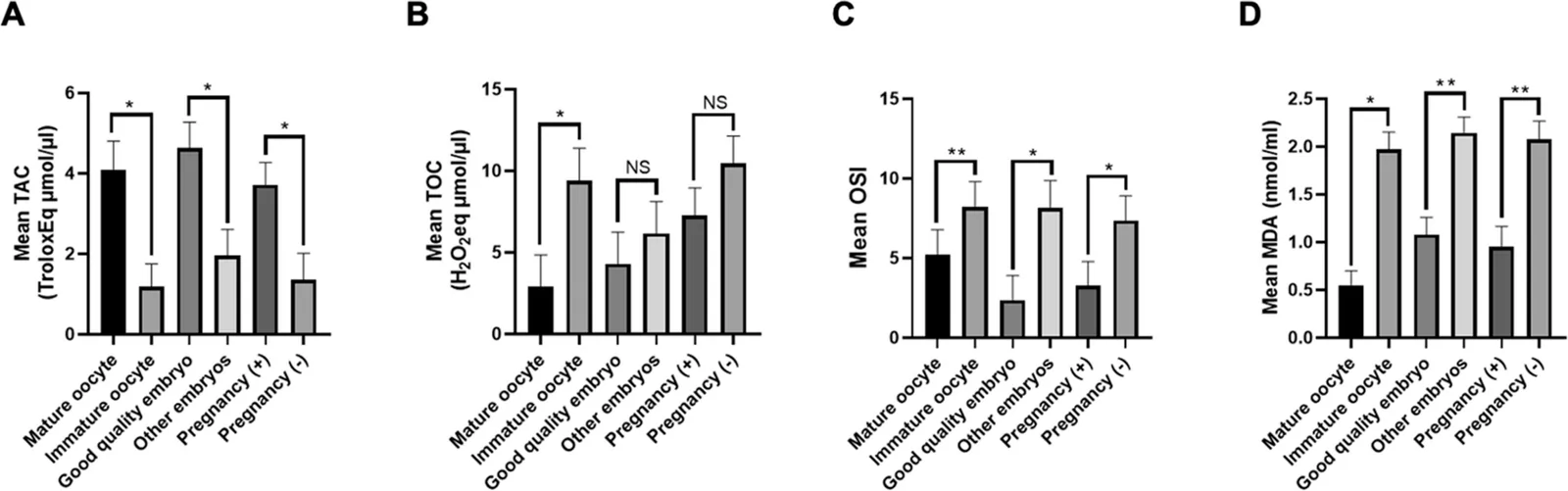
Oxidative stress parameters according to IVF outcomes. Comparative analysis of follicular fluid oxidative stress parameters according to oocyte maturity, embryo quality, and pregnancy outcome. Data are presented as total antioxidant capacity (TAC), total oxidant capacity (TOC), oxidative stress index (OSI), and malondialdehyde (MDA) levels. Values are expressed as mean ± SD. Statistical comparisons were performed using independent samples t-test or Mann–Whitney U test, as appropriate. *p < 0.05 was considered statistically significant; NS, not significant. n represents the number of follicles analyzed per group

We evaluated oxidative stress parameters in follicular fluid according to oocyte maturity, embryo quality, and pregnancy outcome (Fig. 2). Follicles associated with mature oocytes demonstrated significantly higher total antioxidant capacity (TAC) compared with follicles containing immature oocytes (4.09 ± 1.20 vs. 1.13 ± 1.15, *p* < 0.05). Total oxidant capacity (TOC) was significantly lower in mature oocyte-associated follicles than in immature oocyte-associated follicles (2.98 ± 2.12 vs. 9.30 ± 2.21, p < 0.05). Accordingly, oxidative stress index (OSI) values were markedly reduced in mature oocyte follicles (5.20 ± 2.28 vs. 8.20 ± 1.99, *p* < 0.05). Malondialdehyde (MDA) levels were also significantly lower in follicles yielding mature oocytes compared with immature oocytes (0.52 ± 0.74 vs. 1.98 ± 0.86, *p* < 0.05).

When analyzed according to embryo quality, TAC levels were higher in follicles yielding good-quality embryos compared with those yielding other embryos (4.63 ± 1.81 vs. 1.98 ± 1.21, *p* < 0.05). OSI values were significantly lower in the good-quality embryo group (2.26 ± 2.16 vs. 8.10 ± 2.20, *p* < 0.05), while MDA concentrations were also reduced (1.02 ± 0.81 vs. 2.12 ± 0.75, *p* < 0.05). TOC levels did not differ significantly between embryo quality groups.

Similarly, follicles from pregnancy-positive cycles exhibited higher TAC levels compared with pregnancy-negative cycles (3.81 ± 1.11 vs. 1.30 ± 1.03, *p* < 0.05). OSI values were significantly lower in pregnancy-positive cycles (3.33 ± 2.06 vs. 7.26 ± 2.03, *p* < 0.05), and MDA levels were reduced (0.98 ± 0.67 vs. 2.03 ± 0.83, *p* < 0.05). No significant difference was observed in TOC levels according to pregnancy outcome.

### PI3K/AKT/mTOR pathway expression in relation to IVF outcomes

**Fig. 3:**
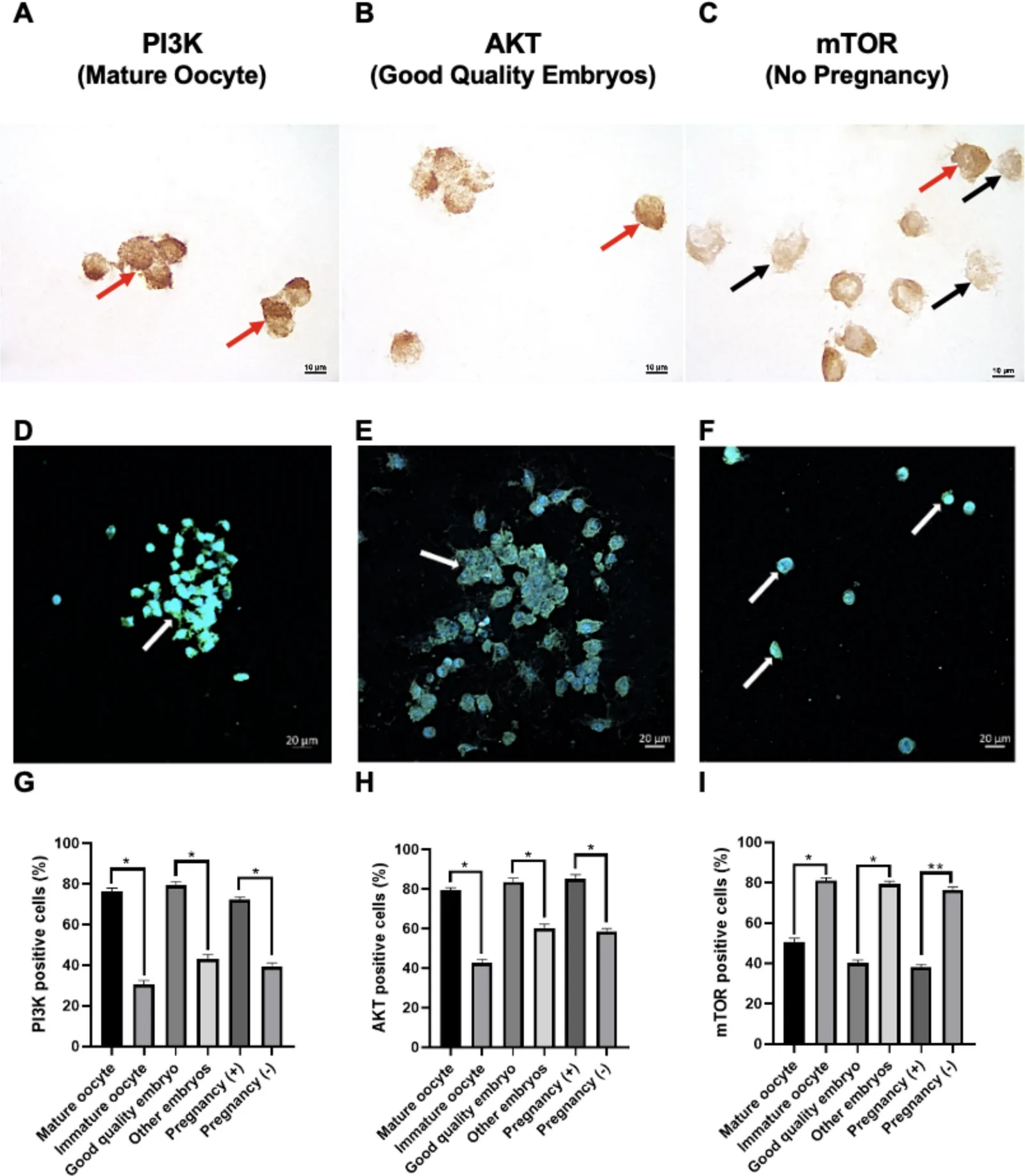
Expression of PI3K, AKT, and mTOR in granulosa cells. (**A**–**C**) Representative light microscopy images showing cytochemical expression of PI3K, AKT, and mTOR in granulosa cells across groups stratified by oocyte maturity, embryo quality, and pregnancy outcome (× 100 magnification). Red arrows indicate positively stained cells, while black arrows indicate weakly stained or negative cells. Scale bar = 10 µm. (D–F) Representative immunofluorescence analysis images demonstrating PI3K, AKT, and mTOR expression patterns in granulosa cells. Scale bar = 20 µm. (**G**–**I**) Quantitative analysis showing the percentage of immunopositive cells for each protein across IVF outcome groups. Values are expressed as mean ± SD. *p < 0.05 was considered statistically significant. n represents the number of follicles analyzed per group

The expression profiles of PTEN/PI3K/AKT/mTOR pathway proteins in granulosa cells were analyzed according to oocyte maturity, embryo quality and pregnancy outcome (Fig. 3).

PI3K expression was significantly higher in granulosa cells associated with mature oocytes compared with immature oocytes (76.82 ± 2.22 vs. 30.66 ± 2.45, *p* < 0.05). Similarly, AKT expression was increased in mature oocyte-associated follicles (79.36 ± 8.21 vs. 42.87 ± 8.91, *p* < 0.05). In contrast, mTOR expression was significantly lower in the mature oocyte group (50.82 ± 6.35 vs. 81.25 ± 6.99, *p* < 0.05).

In follicles yielding good-quality embryos, PI3K expression was significantly higher than in follicles yielding other embryos (79.33 ± 2.19 vs. 43.19 ± 2.68, *p* < 0.05). AKT expression followed a similar pattern (83.82 ± 7.99 vs. 60.21 ± 8.22, *p* < 0.05), whereas mTOR expression was significantly reduced (40.15 ± 6.59 vs. 79.35 ± 6.98, *p* < 0.05).

Pregnancy-positive cycles demonstrated significantly higher PI3K (72.35 ± 2.45 vs. 39.12 ± 2.77, *p* < 0.05) and AKT expression (85.38 ± 8.99 vs. 58.29 ± 11.29, *p* < 0.05), together with significantly lower mTOR expression (38.22 ± 7.21 vs. 76.39 ± 7.82, *p* < 0.05), compared with pregnancy-negative cycles.

No significant differences were detected in PTEN, PDK1, TSC1 or TSC2 expression across any IVF outcome comparisons.

### Follicular fluid hormonal profile in relation to IVF outcomes

**Fig. 4:**
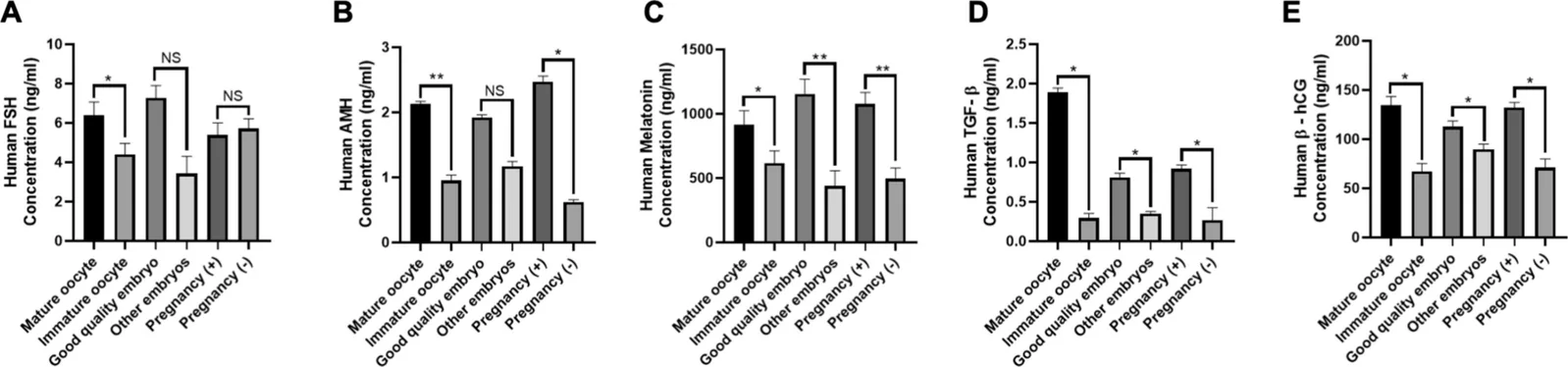
Follicular fluid hormonal profile according to IVF outcomes. Comparative analysis of follicular fluid hormone levels, including follicle-stimulating hormone (FSH), anti-Müllerian hormone (AMH), melatonin, transforming growth factor-β (TGF-β), and β-human chorionic gonadotropin (β-hCG), according to oocyte maturity, embryo quality, and pregnancy outcome. Values are expressed as mean ± SD. Statistical comparisons were performed using independent samples t-test or Mann–Whitney U test, as appropriate. *p < 0.05 was considered statistically significant; NS, not significant. n represents the number of follicles analyzed per group

Follicular fluid concentrations of FSH, AMH, melatonin, TGF-β and β-hCG were analyzed according to oocyte maturity, embryo quality and pregnancy outcome (Fig. 4). Follicular FSH levels were significantly higher in mature oocyte-associated follicles compared with immature oocytes (6.30 ± 1.61 vs. 4.39 ± 1.63, p < 0.05), whereas no significant differences were observed according to embryo quality or pregnancy outcome.

AMH concentrations were significantly elevated in follicles yielding mature oocytes compared with immature oocytes (2.12 ± 0.25 vs. 0.92 ± 0.39, p = 0.002). Additionally, AMH levels were higher in pregnancy-positive cycles than in pregnancy-negative cycles (2.43 ± 0.43 vs. 0.61 ± 0.29, p < 0.05).

Melatonin levels were significantly higher in follicles associated with mature oocytes (912.8 ± 239.23 vs. 618.41 ± 250.26, *p* < 0.05), good-quality embryos (1103.5 ± 243.12 vs. 427.69 ± 222.49, *p* < 0.05) and pregnancy-positive cycles (1036.1 ± 145.32 vs. 495.11 ± 153.94, p < 0.05).

TGF-β concentrations were significantly increased in follicles yielding mature oocytes (1.86 ± 0.32 vs. 0.27 ± 0.21, *p* < 0.05) and good-quality embryos (0.79 ± 0.38 vs. 0.34 ± 0.19, *p* < 0.05), and were also higher in pregnancy-positive cycles compared with negative cycles (0.90 ± 0.28 vs. 0.23 ± 2.45, *p* < 0.05).

The relatively large standard deviation observed in the pregnancy-negative group likely reflects biological heterogeneity as well as the limited sample size.

Similarly, follicular β-hCG levels were significantly higher in mature oocyte-associated follicles (134.5 ± 5.22 vs. 67.96 ± 7.26, *p* < 0.05), good-quality embryo groups (112.7 ± 4.30 vs. 89.76 ± 4.32, *p* < 0.05) and pregnancy-positive cycles (131.4 ± 4.38 vs. 71.06 ± 5.26, *p* < 0.05).

### Granulosa cell apoptosis and chromatin integrity in relation to IVF outcomes

**Fig. 5:**
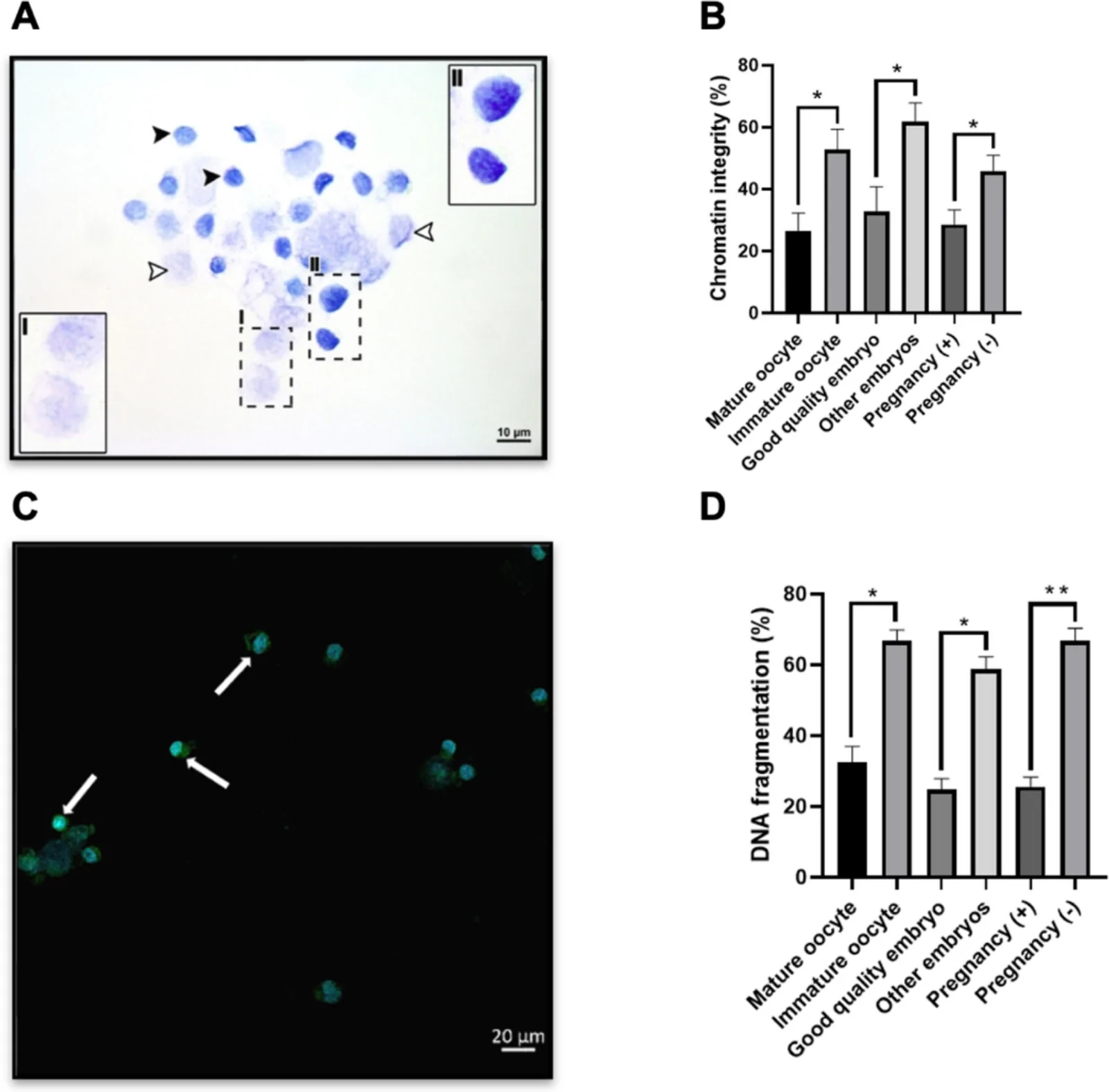
Granulosa cell apoptosis and chromatin integrity. (**A**) Representative light microscopy images of granulosa cells stained with Toluidine Blue (TB). White arrowheads indicate TB-negative cells, while black arrowheads indicate TB-positive cells. Scale bar = 10 µm. (**B**) Higher magnification images illustrating TB-negative (I) and TB-positive (II) granulosa cells. Scale bar = 20 µm. (**C**–**D**) Representative fluorescence microscopy images showing TUNEL-positive granulosa cells (× 20 magnification). White arrows indicate TUNEL-positive nuclei. Quantitative analysis of DNA fragmentation and chromatin integrity is presented as mean ± SD. *p < 0.05 was considered statistically significant. n represents the number of follicles analyzed per group

Granulosa cell apoptosis and chromatin integrity were evaluated using DNA fragmentation analysis and toluidine blue staining, and findings were compared according to oocyte maturity, embryo quality and pregnancy outcome (Fig. 5). Granulosa cells obtained from follicles associated with mature oocytes exhibited significantly lower DNA fragmentation rates compared with those from immature oocytes (32 ± 4.12% vs. 66 ± 2.17%, *p* < 0.05). Similarly, DNA fragmentation was reduced in follicles yielding good-quality embryos (24 ± 2.54% vs. 58 ± 3.12%, *p* < 0.05) and in pregnancy-positive cycles (25 ± 2.29% vs. 66 ± 3.26%, *p* < 0.05).

Chromatin integrity analysis demonstrated significantly lower proportions of toluidine blue–positive cells in mature oocyte-associated follicles compared with immature oocytes (26 ± 6.21% vs. 53 ± 8.33%, *p* < 0.05). Improved chromatin integrity was also observed in follicles yielding good-quality embryos (33 ± 9.38% vs. 62 ± 6.09%, *p* < 0.05) and in pregnancy-positive cycles (28 ± 5.15% vs. 45 ± 5.22%, *p* < 0.05).

Overall, the observed differences were associated with moderate to large effect sizes across key comparisons, supporting the biological relevance of the findings despite the limited sample size.

## Discussion

The present study provides a comprehensive evaluation of multiple follicular dimensions in relation to IVF outcomes at the individual oocyte level. The findings indicate consistent associations across oxidative stress parameters, hormonal mediators, granulosa cell integrity, and PI3K/AKT/mTOR signaling, suggesting that oocyte developmental competence reflects a coordinated follicular microenvironment rather than a single dominant factor.

One of the most consistent findings of this study was the association between favorable IVF outcomes and reduced oxidative stress within the follicular fluid. Lower OSI and MDA levels across mature oocytes, good-quality embryos and pregnancy-positive cycles suggest a protective oxidative balance in competent follicles. Although physiological ROS generation is essential for ovulation and normal follicular function, excessive oxidative stress has been shown to disrupt meiotic spindle formation, mitochondrial activity and DNA integrity [27, 28]. Previous studies investigating oxidative stress in follicular fluid have reported heterogeneous results, likely reflecting differences in patient populations, stimulation protocols and outcome definitions [29–34]. Our findings indicate that oxidative stress parameters should not be interpreted in isolation but rather as part of an integrated follicular environment influencing oocyte competence.

These findings should be interpreted with caution, as oxidative stress in the present study was evaluated using indirect biochemical markers rather than direct measurements of intracellular ROS or mitochondrial function. Therefore, the results reflect the overall oxidative status of the follicular environment rather than precise ROS dynamics or cellular redox regulation.

In parallel with oxidative balance, granulosa cell integrity emerged as a key component associated with oocyte developmental potential. Both DNA fragmentation and impaired chromatin integrity were significantly reduced in follicles associated with favorable IVF outcomes. Apoptosis in granulosa and cumulus cells has previously been linked to compromised oocyte maturation, reduced embryo quality and lower pregnancy rates [35–37]. The concordance between TUNEL and Toluidine Blue findings in the present study further supports the concept that preserved granulosa cell genomic integrity reflects a supportive follicular milieu conducive to oocyte competence.

It should also be noted that granulosa cell analyses were based on manual evaluation of a limited number of cells per sample, which may affect quantitative robustness and introduce observer-related variability. The absence of replicate measurements and automated image analysis represents an additional limitation. Future studies incorporating larger cell counts, inter-observer validation, and automated quantification methods are needed to enhance reproducibility and analytical precision.

A central molecular finding of this study was the coordinated modulation of the PTEN/PI3K/AKT/mTOR signaling pathway in granulosa cells. Increased PI3K and AKT expression, accompanied by suppressed mTOR activity, was consistently observed in follicles associated with mature oocytes, good-quality embryos and pregnancy. The PI3K/AKT pathway is a critical regulator of follicular activation, granulosa cell survival and oocyte meiotic progression [14, 38, 39]. While previous human studies have reported altered PI3K/AKT-related gene expression in cumulus cells associated with IVF outcomes [37, 40], results have not been uniform, possibly due to differences in analytical approaches and outcome parameters. Our findings suggest that activation of PI3K/AKT signaling, together with relative suppression of mTOR, may represent a favorable signaling configuration rather than isolated pathway activation.

This pattern likely reflects context-dependent mTOR regulation rather than canonical linear pathway activation. Accordingly, these findings should not be interpreted as indicating a universally beneficial effect of mTOR suppression. Moreover, the absence of phosphorylation analysis and downstream functional assessment limits mechanistic interpretation of pathway activity.

Interestingly, PTEN, TSC1 and TSC2 expression levels did not differ significantly between outcome groups. Given that PTEN and TSC proteins are well-established negative regulators of the PI3K/AKT/mTOR axis [14, 41, 42], their stability in our study suggests that mTOR modulation may occur through alternative regulatory mechanisms or pathway crosstalk rather than classical upstream inhibition. Crosstalk between PI3K/AKT signaling, oxidative stress responses and DNA damage pathways has been increasingly recognized [43], and such interactions may contribute to the signaling patterns observed in competent follicles.

The hormonal composition of follicular fluid further contributed to the multidimensional profile associated with oocyte competence. In the present study, follicular FSH and AMH levels were primarily associated with oocyte maturity, whereas melatonin, TGF-β and β-hCG levels showed broader associations with embryo quality and pregnancy outcomes. FSH and AMH are well-established indicators of follicular development and ovarian reserve, yet their follicular concentrations appear to reflect maturation rather than downstream developmental competence [44, 45]. In contrast, melatonin has been widely reported to exert antioxidant and cytoprotective effects within the follicle, improving oocyte quality and embryo development [46, 47]. Similarly, TGF-β plays a role in follicular growth, oocyte maturation and implantation processes, while follicular β-hCG has been suggested to reflect follicular differentiation and luteinization status [48, 49]. Our findings support the notion that these hormonal mediators act synergistically within the follicular microenvironment rather than functioning as isolated indicators. The variability observed in certain parameters, particularly TGF-β levels, may reflect underlying biological heterogeneity within the study population as well as the limited sample size.

Accordingly, these associations should be interpreted with caution, as the present analysis did not include adjustment for potential confounding factors such as patient age, ovarian stimulation parameters, or follicle size, nor did it incorporate parallel serum hormone measurements. Therefore, the observed relationships reflect intra-follicular patterns rather than independent or systemic hormonal effects.

Overall, the convergence of reduced oxidative stress, preserved granulosa cell integrity, coordinated PI3K/AKT/mTOR signaling and a supportive hormonal environment underscores the multifactorial nature of oocyte developmental competence. Rather than proposing a single biomarker for oocyte selection, this study supports a panel-based and pattern-oriented interpretation in which multiple follicular dimensions collectively reflect oocyte quality. Importantly, the present findings do not establish a predictive model but instead highlight coordinated biological patterns that may inform future multivariate and predictive analyses.

Given the multidimensional and exploratory design of the study, multiple statistical comparisons were performed without formal adjustment for multiple testing. This approach was adopted to facilitate the identification of coordinated biological patterns while minimizing type II error.

However, this strategy may increase the risk of type I error. Therefore, the results should be interpreted with caution and considered as hypothesis-generating rather than confirmatory findings. Future studies with larger sample sizes and appropriate statistical correction methods are warranted to validate these observations.

This study has several limitations that should be considered. First, the relatively small sample size and the inclusion of a limited number of patients may restrict the generalizability of the findings. Although analyses were performed at the individual follicle–oocyte level to capture biological variability, the non-independence of follicles originating from the same patient may introduce intra-patient clustering and potential pseudo-replication bias. In this context, the absence of mixed-effects or hierarchical modeling represents a methodological limitation. This approach was primarily intended to identify biological patterns rather than to establish independent statistical inference at the patient level.

Second, due to the exploratory and multidimensional nature of the study, no a priori sample size calculation or adjustment for multiple comparisons was performed. Therefore, the findings should be interpreted as hypothesis-generating associations rather than definitive predictive relationships.

Another important limitation is the absence of functional validation experiments. The present findings are based on correlative associations and do not establish causal relationships. Functional studies, including pathway modulation and in vitro mechanistic analyses, are required to confirm the biological roles of the identified pathways.

Future studies with larger, independent cohorts and appropriate multivariate modeling are needed to validate the observed patterns and to assess their potential clinical applicability.

Although the present findings provide insight into the multidimensional follicular environment associated with oocyte competence, they do not establish a clinically applicable prediction model or define actionable thresholds. Therefore, the results should be interpreted as hypothesis-generating rather than directly translatable to clinical decision-making. Further studies incorporating validation cohorts and predictive modeling are required to determine the potential clinical utility of these findings.

Importantly, although the multidimensional nature of follicular regulation is well recognized, the present study contributes by integrating multiple biological domains within the same follicular context, providing a more cohesive interpretation of oocyte competence at the microenvironmental level.

In conclusion, the findings suggest that oocyte developmental competence in IVF is associated with a coordinated follicular microenvironment rather than a single isolated determinant. Favorable IVF outcomes were characterized by reduced oxidative stress, preserved granulosa cell chromatin integrity and survival, a supportive hormonal milieu, and a distinct modulation of the PTEN/PI3K/AKT/mTOR signaling axis. Collectively, these findings support a multidimensional, panel-based interpretative approach rather than a predictive framework for understanding oocyte developmental competence and highlight the value of integrated follicular assessment in assisted reproductive technologies.

## Data Availability

The datasets generated and/or analyzed during the current study are available from the corresponding author on reasonable request.
